# Awareness Campaigns to Prevent Diabetic Ketoacidosis at Diabetes Onset Are Successful When Constantly Maintained: From Local to Federal State Results

**DOI:** 10.1155/pedi/5491154

**Published:** 2025-08-19

**Authors:** Martin Holder, Jacqueline Weiler, Reinhard W. Holl, Stefan Ehehalt

**Affiliations:** ^1^Pediatric Endocrinology and Diabetology, Klinikum Stuttgart-Olgahospital, Stuttgart, Germany; ^2^Public Health Department of Stuttgart, Stuttgart, Germany; ^3^Institute of Epidemiology and Medical Biometry, CAQM, Ulm University, Ulm, Germany

## Abstract

**Objective:** To expand the effective local Stuttgart childhood diabetic ketoacidosis (DKA) prevention campaign to the federal state of Baden-Württemberg (BW) in Germany.

**Research Design and Methods:** All public health departments (PHDs) in BW were invited to participate. The DKA-incidence at diabetes onset was compared between participating and nonparticipating districts, prior (2015–2020) and during the campaign (2021–2023).

**Results:** A total of 3038 children and adolescents were newly diagnosed with type 1 diabetes in BW during the observation period. DKA was present in 990 children (32.6%), severe DKA in 346 (11.4%). In total 14 of 38 PHD (37%) participated. DKA rates increased both in participating (29.9%–36.3%) and in nonparticipating districts (27.0%–41.0%; *p* < 0.0001 for time-trend). However, there was a significant interaction between time-interval and the groups of districts (*p* < 0.03) reflecting a significant treatment effect in the intervention group.

**Conclusions:** The expansion of our local awareness campaign was possible and successful.

## 1. Introduction

Awareness campaigns like the Stuttgart Ketoacidosis awareness campaign informing parents and physicians about the typical clinical symptoms of type 1 diabetes can significantly reduce the risk for diabetic ketoacidosis (DKA) at diabetes onset [1, 2]. The prevention campaigns to date have been carried out in very different countries or different geographic areas of countries, have different target groups and use different information materials [2]. Our aim was to determine, if the outcomes of the effective local Stuttgart campaign, can also be expanded to the larger area of the federal state of Baden-Württemberg (BW) in Germany.

## 2. Materials and Methods

**Figure 1:**
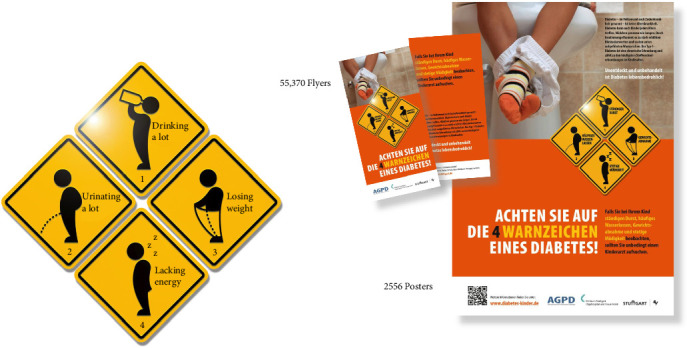
Information materials, which were used during the campaign. In particular, information icons about the four typical clinical symptoms of type 1 diabetes in more detail.

The federal state of BW is located in the southwest of Germany. With almost 12 million inhabitants it is the third largest state in Germany. There are 38 public health departments (PHDs) covering the state. All PHDs were informed and invited to take part in the campaign, which started in 2021. Information flyers and posters illustrating the typical symptoms of diabetes were distributed at the school entry health examinations as described for the local campaign previously [1, Figure 1]. In addition, daycare centers and pediatric practices were involved at the respective district level and regular public relations work was maintained. In total 14 of 38 PHD (37%) participated in the intervention. The awareness campaign was continued for 3 years (2021–2023). 2015–2020 was chosen as the reference period. During the campaign 55,370 information flyers and 2556 posters were distributed. Figure 1 shows the information materials, which were used during the campaign. In particular, information icons depict the four typical clinical symptoms of type 1 diabetes in more detail: drinking a lot, urinating a lot, losing weight, and lacking energy (polydipsia, polyurea, weight loss, and fatigue).

We used data from the German prospective diabetes follow-up registry (DPV registry) of children and adolescents aged 0.5–16 years at diagnosis of type 1 diabetes between January 1, 2015 and December 31, 2023. Data are documented from all participating 28 pediatric diabetes care institutions in BW using the standardized DPV documentation software [3]. The ethics committee of Ulm University (Ulm, Germany) approved the analysis of anonymized data from the DPV registry (ethics approval 314/21); local review boards approved data collection.

Demographic data included age at diabetes onset, sex, HbA1c, BMI SDS values, immigrant background (patient or at least one parent born outside of Germany), and the reference group. DKA was defined as pH < 7.3 and/or serum bicarbonate < 18 mmol/L and severe DKA as pH < 7.1 and/or bicarbonate < 5 mmol/L [4, 5]. Based on the ZIP-code of their home at diabetes onset, children were assigned to their respective PHD, which were classified as participating or nonparticipating based on feedback to the study organizers.

Continuous variables are presented as median and upper and lower quartile, binary variables as proportion. Wilcoxon rank-sum test for continuous variables and *X*^2^ test for proportions were used for unadjusted group comparisons, with Bonferroni-Holm correction for multiple tests. Logistic regression models were used with onset period (2015–2020 and 2021–2023), living in a district with or without participation in the campaign, gender and age-group (0.5–16, < 10 years) as independent variables. In addition to these confounders, an interaction term for onset period with district participation was added.

A two-sided *p*-value < 0.05 was considered statistically significant. All analyses were performed with SAS version 9.4 (build TS1M7, SAS Institute Inc., Cary, NC,USA) on a Windows Server mainframe.

## 3. Results

During the entire observation period (2015–2023) 3038 children and adolescents were newly diagnosed with type 1 diabetes. DKA at diabetes onset was present in 990 children (32.6%), severe DKA in 346 (11.4%).  Table 1 provides an overview of the cohort. During the observation period, the prevalence of DKA increased in both cohorts (*p* < 0.0001) when comparing the precampaign (2015–2020) to the campaign periods (2021–2023).

**Figure 2:**
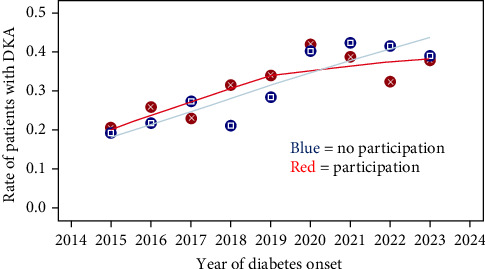
Overview about the annual DKA rates on diabetes onset separately for participation of PHD (districts) or not.

As this trend continues during the campaign in the nonparticipating districts (PHD), the increase of DKA rates were less pronounced in the participating districts. Prior to the campaign, PHDs (districts) who participated in the campaign had comparable DKA rates before the campaign as PHDs (districts) who did not participate (29.9% vs. 27%). During the campaign, DKA increased less in the participating versus nonparticipating PHDs (36.3% vs. 41.0%). There was a significant interaction-term between onset period and participation of PHD districts reflecting the effectiveness of the campaign (*p*=0.03) (Table 2, Figure 2). Analyzes from children < 10 years and from children and adolescents with severe DKA showed similar, but not significant results. At the end of the intervention we conducted an online survey with all participating PHDs. The overall response to the campaign was positive: 63% of respondents said it was an effective, accessible way of sharing information about a relevant health topic with children at the time of their school entry examination. Overall, 75% of respondents felt that the messages were clear and easy to understand. Overall, 67% of respondents said that implementing the campaign did not lead to an increased workload due to more enquiries from concerned citizens. Overall, 70% of respondents wanted to continue providing information about the symptoms of diabetes, particularly as part of school entry examinations, via the parents' guide or as part of events to mark World Diabetes Day. The survey showed that the campaign was pursued most actively during the first 2 years. Analysis of the first 2 years of the campaign (2021–2022, total 2684 manifestations of diabetes) in comparison to 2015–2020 showed comparable results: 29.8% versus 27.1% DKA-rates of participating and nonpaticipating PHD before and 35.6% versus 41.3% during the campaign. Again the interaction term “campaign period” with “PHD participation” was significant (*p* < 0.04, Table 2).

## 4. Discussion

Awareness campaigns about the typical clinical symptoms of type 1 diabetes can result in a significant reduction of DKA at diabetes onset [1, 2]. Following the successful implementation of the Stuttgart Ketoacidosis Prevention Campaign at the local level, we have extended this proven concept to the entire federal state of BW in Germany. The question arose as to whether the expansion was possible and feasible.

Our objective was to replicate the intervention from the previous local campaign. With this campaign, we were also able to achieve a significant reduction in the DKA rates.

In Italy, around the millenium, a significant reduction in DKA over 65% could be achieved by prevention program over 8 years in schools and private practices [6]. A recent systematic review shows, that the frequency of DKA at diabetes onset is still high in Italy because of the delay in diagnosis due to lack of awareness among parents, other caregivers, schoolteachers, or healthcare professionals about the symptoms of type 1 diabetes in children [7].

In Australia, where a similar campaign to ours was conducted, a reduction of DKA of almost 25% was achieved. Education material and posters about the typical symptoms of type 1 diabetes and blood sugar and ketone test stripes were distributed to day-care centers, schools and pediatric practices. In the control group there was no change in DKA incidence at initial manifestation over these 2 years (37.4% and 38.6%) [8].

The Canadian Diabetes Association initiated in September 2024 the DKA prevention campaign “Recognizing the Signs of Diabetes in Children at School.” In contrast to our campaign the target population are children and adolescents in schools, school staff, parents, and caregivers. Specific school material was developed and circulated among health care professional organizations across Canada [9].

However, not all awareness campaigns from the literature were successful. In Austria, using a community-based, poster-focused prevention program over 2 years, there was no reduction in the frequency of DKA [10]. In comparison to our campaign, the Austrian campaign was conducted only over 2 years.

Similar to reports from several countries worldwide, we observed increasing DKA-rates in both cohorts during the observation period. This marked exacerbation of the preexisting increase in DKA prevalence was due to COVID-19 pandemic [11]. This finding highlights the need for early and timely diagnosis of type 1 diabetes in children and adolescents.

Antibodies screening of the general population can help to reduce DKA frequency at disease onset and allow interventions to prevent or postpone the disease [12, 13]. In addition, diabetes awareness campaigns are an effective and complementary tool for lowering DKA rates. A current systematic review could show that awareness campaigns can also improve other parameters, such as acute complications, HbA1c and C-peptide levels, length of hospitalization, and cost [7]. To be effective, campaigns must follow specific principles for target population, modality, and minimal duration. In our campaign, the specific target population were families with young children and healthcare professionals. Healthcare professionals must intervene rapidly when parents report symptoms; therefore, the targeted message is that the earlier the diagnosis, the lower the frequency of DKA, particularly in younger children. It is important for the success of the campaign that the target population has seen, read, and understood the posters and flyers with information about DKA and diabetes diagnosis. We have been able to integrate this information well into school entry health assessments.

Campaigns should last at least 2 years and must be renewed every 5 years to maintain their effectiveness [7]. We conducted our campaign over 3 years but the efforts weakened in some PHDs in the 3rd year. The major weakness of our campaign is that only 14 of 38 PHD (37%) participated. On the other hand, this allowed to use nonparticipating districts as controls. The strengths of our campaign were the modalities: a well-known, simple, effective, and proven information strategy, mandatory examinations for all children and their families of every birth cohort and clear contact persons in the PHD.

## 5. Conclusions

The expansion of our local awareness campaign throughout the federal state of BW in Germany was possible and successful. This means that the prevention campaign presented here can effectively and significantly reduce DKA at diabetes onset. The combination of a personal approach to the respective players and cross-sectoral, interdisciplinary cooperation (health authorities, pediatric and adolescent practices, hospitals, daycare centers, and schools) is an essential factor for the feasibility and successful implementation of prevention campaigns. In order to achieve sustainable success, the campaign must be repeated (at longer intervals) or carried out continuously with the same intensity. Awareness campaigns need ongoing efforts to provide continued preventive effects.

## Figures and Tables

**Table 1:** Descriptive characteristics of all children and adolescents with type 1 diabetes during the observation period and separately by DKA at diabetes onset.

Variable	Total observation period (2015–2023)	No DKA	With DKA
Patients, *N*	3038	2048	990
Males, *N* (%)	1664 (54.8)	1157 (65.5)	507 (51.3)
Age (years) and mean (interquartile range)	8.8 (5.3–12.2)	8.9 (5.6–12.3)	8.5 (4.9–12.0)
HbA1c (%) and mean (interquartile range)	11.6 (10.1–13.2)	11.2 (9.7–12.8)	12.3 (11.0–13.7)
BMI-SDS, RKI-KIGGS-Daten, and mean (interquartile range)	−0.49 (−1.25–0.37)	−0.40 (−1.20–0.39)	−0.51 (−1.38–0.35)
Immigrant background, *N* (%)	950 (31.3)	593 (29.0)	355 (35.9)
DKA at diabetes onset, *N* (%)	990 (32.6)	—	—
Severe DKA at diabetes onset, *N* (%)	346 (11.4)	—	—

*Note:* pH < 7.3 and/or serum bicarbonate < 18 mmol/L at treatment initiation. Severe DKA: pH < 7.1 and/or serum bicarbonate < 5 mmol/L RKI-KIGGS = National reference dataset. The German Health Interview and Examination Survey for Children and Adolescents (KiGGS) from the Robert Koch Institute (RKI) regularly collects data on various aspects of children's and adolescents' health and thus enables a population-representative description of the health on in Germany.

Abbreviation: DKA, diabetic ketoacidosis.

**Table 2:** Comparison of DKA-rates before campaign (2015–2020) with DKA-rates during campaign (2021–2023) adjusted whether PHDs (districts) have participated or not.

PHD (district)	Participation	No participation	*p*-Value
DKA-rate before campaign (%) (2015–2020)	29.9	27.0	NS

DKA-rate during campaign (%) (2021–2023)	36.3	41.0	< 0.0001*⁣*^*∗*^ 0.03*⁣*^*∗∗*^

The first 2 years of the campaign (2021 and 2022)	35.6	41.3	0.04*⁣*^*∗∗*^

Abbreviations: DKA, diabetic ketoacidosis; NS, nonsignificant; PHD, public health department.

⁣^*∗*^For comparison between 2015–2020 and 2021–2023 (time-trend).

*⁣* ^*∗∗*^For interaction between time-interval and the groups of districts (PHDs).

## Data Availability

The data that support the findings of this study are available from the corresponding author upon reasonable request.

## Nomenclature

DKA: Diabetic ketoacidosis
PHD: Public Health Department.
